# Cost-effectiveness of glaucoma screening in cataract camps versus opportunistic and passive screening in urban India: A study protocol

**DOI:** 10.12688/f1000research.17582.3

**Published:** 2019-04-29

**Authors:** Shalinder Sabherwal, Denny John, Suneeta Dubey, Saptarshi Mukherjee, Geetha R. Menon, Atanu Majumdar

**Affiliations:** 1 Community Ophthalmology, Shroff's Charity Eye Hospital, New Delhi, Delhi, 110002, India; 2 Evidence Synthesis, Campbell Collaboration, New Delhi, Delhi, 110070, India; 3 Glaucoma Services, Shroff's Charity Eye Hospital, New Delhi, Delhi, 110002, India; 4 Department of Optometry, Shroff's Charity Eye Hospital, New Delhi, Delhi, 110002, India; 5 National Institute of Medical Statistics, Indian Council of Medical Research, New Delhi, Delhi, 110029, India; 6 Department of Statistics, Shroff's Charity Eye hospital, New Delhi, Delhi, 110002, India

**Keywords:** screening, glaucoma, costs, cost-effectiveness, screening, detection rate

## Abstract

India has an estimated 12 million people affected with glaucoma; however, no organised screening programme exists. Cases are usually detected opportunistically. This study documents the protocol for detecting glaucoma in suspects in cataract camps conducted by Shroff Charity Eye Hospital in North India. We report a cost-effectiveness alongside prospective study design of patients attending cataract camps where glaucoma screening will be integrated. The eligible population for glaucoma screening is non-cataract patients. Patients will undergo glaucoma screening by a trained optometrist using a pre-determined glaucoma screening algorithm. Specific diagnostic cut-off points will be used to identify glaucoma suspects. Suspected patients will be referred to the main hospital for confirmatory diagnosis and treatment. This group will be compared to a cohort of patients arriving from cataract camps conducted by the institute in similar areas and undergoing examination in the hospital. The third arm of the study includes patients arriving directly to the hospital for the first time. Cost data will be captured from both the screening components of cataract-only and glaucoma screening-integrated camps for screening invitation and screening costs. For all three arms, examination and treatment costs will be captured using bottom-up costing methods at the hospital. Detection rates will be calculated by dividing the number of new cases identified during the study by total number of cases examined. Median, average and range of costs across the three arms will be calculated for cost comparisons. Finally, cost-effectiveness analysis will be conducted comparing cost per case detected across the three arms from a quasi-societal perspective with a time horizon of 1 year **. **Ethics approval for the study has been obtained from the institutional ethics committee of the hospital. The study protocol will be useful for researchers and practitioners for conducting similar economic evaluation studies in their context.

## Introduction

The term ‘glaucoma’ was derived from the Greek word γλαύκωμα (Glaucosis) during the Hippocratic era of 400 BC, and it meant the greenish pupillary hue in eye that is much different from the normal pupillary color ^1^. The two main types of glaucoma are angle-closure glaucoma (ACG) and open-angle glaucoma (OAG). In ACG, pupillary blockage leads to sudden rise of intraocular pressure (IOP) manifesting as severe headache, intense ocular pain, redness, clouding or haziness of cornea, and are further compounded with acute and marked diminution or even loss of vision within next few hours, thus presenting as an emergency and warranting immediate therapeutic intervention to save vision. In contrasts, OAG remains silent for several years till the slow and gradual rise in IOP leads to manifest optic nerve damage and peripheral visual fields changes, and can be diagnosed in ocular examination or linked with the family history.

Glaucoma is the second commonest cause of blindness in the world ^2^. There are estimated 12 million people affected by glaucoma in India ^3^. The two main types of glaucoma are angle-closure glaucoma (ACG) and open-angle glaucoma (OAG). It is estimated that about 70% of OAG and 80% of ACG cases occur in developing nations ^4^. In India it is estimated that primary OAG (POAG) affects around 6.48 million people and primary ACG (PACG) affects around 2.54 million ^3^. In India, it is estimated that among the 40+ age group, every eighth person could be at risk of, or, suffering from glaucoma ^3^. As there is no organised screening for glaucoma, opportunistic case finding is the commonest method for case detection. It has been shown that around 90% of the glaucoma remains undiagnosed in both rural and urban populations in India ^5, 6^. Another study found that only 50% of people with glaucoma ever visited an ophthalmologist ^7^. Thus, stressing the need of a more effective screening program

The use of intraocular pressure (IOP), field testing and optic disc findings for diagnosis of glaucoma has been stressed for effective screening program for identifying glaucoma in the community. It has been shown that none of them individually have positive predictive value or the sensitivity to be used for community-based screening. These tests when used together have much better sensitivity to diagnose early glaucoma.

In most of the developing countries, there is a shortage of ophthalmologists. In India, the availability of ophthalmologists in rural setting is low ^8^. In order for an effective screening program to function there is need for including other ophthalmic personnel such as optometrists and ophthalmic assistants. It has been shown that trained ophthalmic assistants can be effective in detecting glaucoma in community settings ^9^. Thus, the equipment used should be selected according to the cadre involved in screening.

IOP is an important risk factor for developing glaucoma. Although used alone it has a sensitivity of only 47.1%, and specificity of 92.4% if a cut-off of more than 21 mmHg is used for diagnosing POAG ^10^. Different types of tonometers are used to measure IOP. Tonometers based on rebound technology have been found to have accuracy similar to applanation tonometry ^11^. The rebound technology is based on the rebound measuring principle in which a light-weight probe is used to make a momentary contact with the cornea. A software analyzes deceleration and contact time of the probe when it touches the cornea. Deceleration and contact time of the probe change as a function of IOP. An optic disc examination for the cup to disc ratio is used to diagnose glaucoma. Direct ophthalmoscope is relatively inexpensive and portable equipment which has been used in the community setting ^12^. It’s sensitivity has been shown to be around 59% ^13^. Frequency doubling technology (FDT) perimetry has emerged as a quick and inexpensive alternative. The sensitivity of the test has been shown to be around 50% in some studies ^14^ as compared to more than 95% for automated perimetry ^15^. Gonioscopy is considered gold standard for identifying eyes at risk of angle closure but it requires clinical expertise to conduct and interpret the test. Thus, it is not an appropriate screening test ^16^. Van Herick test for peripheral anterior chamber depth (ACD) has been shown to have a sensitivity of 91% for detecting shallow chambers ^17^ and of 61.9% in detecting occludable angles ^18^.

WHO Vision 2020 has prioritised interventions for five causes of avoidable blindness ^19^. Cataract being the commonest cause of avoidable blindness in India has been prioritised by National Program for Control of Blindness. There are regular cataract screening camps organised by both government and non-government service providers. Within these camps the focus is to examine the maximum number of patients over 50 years of age. This research protocol aims to study the costs, cost-effectiveness and detection rate of glaucoma cases in cataract screening camps in North India.

## Methods

### Intervention

Dr. Shroff’s Charity Eye Hospital (SCEH), based in New Delhi, is a tertiary referral center providing general and subspeciality services and training. SCEH conducts one-day cataract screening clinics in villages closer to the city of New Delhi. This is made possible through the financial support of private donors. These programs target patients who have not sought out care due to limited or non-existent local eye care facilities, financial constraints, or in most case a lack of awareness regarding eye-care. In anticipation of the one-day screening clinics, local organizers are responsible for advertising the clinic (e.g. through posters, flyers, or door-to-door visits), managing volunteers and procuring an appropriate facility. The cost of transportation of patients to SCEH is borne by hospital These camps are managed by Program Manager (Outreach) based at SCEH with regards to planning, monitoring, and strategy.

### Rationale for the study

In our data of the camps conducted by the outreach team in Delhi, between June 2016 and May 2017, out of 8283 population of ≥40 years age screened, only 0.25% (n=21) were identified as glaucoma suspects. This is well below the prevalence (1.6–3.5%) found in the rural and urban studies in India ^20^. Model based studies of community screening for glaucoma in rural and urban India has been shown to be cost-effective ^21, 22^. The rationale of this study is to analyse the cost and detection rate of detecting glaucoma in cataract camps. If found to be cost effective, this model can be scaled up for detecting glaucoma suspects from cataract screening camps and referring them for further investigations and management. 

### Screening protocols in camps

In addition to the original team for the outreach camp, one of the trained optometrists, a vision technician for handling FDT and a counsellor would travel for the interventional camps.

All people of age ≥40 years, not detected as having a cataract would undergo screening for glaucoma by trained optometrist using additional equipment. Those detected with cataracts are not included in the study as they would have undergone glaucoma screening as a routine before being advised surgery. Those included in the study would have already undergone vision testing and refraction if indicated. The registration number given at the start of the camp would be recorded by the counsellor. There would be two stations for glaucoma screening. The trained optometrist would carry out disc examination, ACD examination and IOP recording. At the second station FDT would be carried out. Any result beyond cut-off and requiring further intervention would be flagged off. After that the person screened would be received by the counsellor. The test used for screening would be disc examination, FDT and IOP using (Icare) rebound perimetry.

***Training.*** There will be two optometrists trained for the study. The optometrists selected would already be carrying out refractions, basic history taking and slit-lamp examination. The curriculum would pertain to glaucoma-related history-taking and slit-lamp examination. Specifically, for the study the following training would be included for various test to be conducted in the study

### Intraocular Pressure (IOP)

The optometrists would have explained to them the need to calibrate before starting measurements and explained the process of calibration. The principles of avoiding injury while recording IOP would be discussed. The cut-offs being used in the study would be stressed upon

### Cup-to-disc ratio (C/D)

The optometrists would be trained to use direct ophthalmoscope and focus on the disc. They would be exposed to patients and photographs with a variety of disc findings.

### Anterior Chamber Depth (ACD)

The concept of the Van Hericks test would be explained. Although they would be already using a slit-lamp, they would be trained to use a hand-held slit lamp. The cut-offs to be used would be explained. The optometrists would be maintaining a log-book to record their findings, and these would be checked and signed by a glaucoma consultant or a senior fellow.

### Frequency Doubling Technology (FDT)

To carry out a field test with FDT, the optometrists would be trained in handling the equipment, giving instructions to the patients and commenting on the printouts keeping the cut-offs of the study in mind.

The training would be conducted in a classroom with visual presentations for theory. A senior glaucoma fellow ophthalmologist would be involved to take classes with a problem-based approach. Theory class would be scheduled for 1 hour in every working day for 2 weeks. A total of 12 hours would be spent in theory classes. The topics to be covered are mentioned in Table 1.

**Table 1.** Training components for optometrists.

Topic	Activities	Responsibility
Detailed History taking	Glaucoma related history and old report reading	Senior Optometrist (more than 5 years’ experience)
Slit lamp evaluation overview	Detailed Adnexa and Anterior segment evaluation	Senior Optometrist (as above)
Angle Evaluation	Van Herick method	Senior Optometrist
Intra ocular pressure	Icare,	Senior Optometrist
Optic Nerve Head (ONH) evaluation	Cup disc ratio, Disc Size, shape with direct ophthalmoscope	Fellow Glaucoma ophthalmologist

Junior glaucoma consultant would be involved in imparting practical training. In total, 30 h would be taken to develop their practical skills for each trainee. A log book record would be maintained for the patients examined. The criteria used for assessing the level of training are highlighted in Table 2.

**Table 2.** Guidelines used for assessing the levels in training.

Grade	Activities/evaluation	Criteria
Competent	• IOP • Van Herick method • ONH	• Accurate finding
Advance Beginner (need more practice)	• IOP • Van Herick method • ONH	• Accurate finding • Within the range • Can identify glaucomatous change
Beginner (need re-training)	• IOP • Van Herick method • ONH	• Within the range • Can differentiate close and open, but cannot do grading • Cannot report C/D ratio accurately

IOP, intraocular pressure; ONH, optic nerve head.

## Validation

This would be carried out after the training is complete. A total of 30 cases each would be given to both optometrists for recording IOP, ACD and C/D ratio. These would be either new cases or they would be masked to previous records. A senior optometrist would independently record IOP and a glaucoma consultant would examine for ACD and C/D ratio. Both the senior optometrist and glaucoma consultant would be masked to the trainee optometrists’ findings. Sensitivity and specificity would be calculated keeping the trainers as gold standard. If not found to be within an acceptable range (85% sensitivity, and 90% specificity), the training would be repeated for the concerned trainee in the identified area of weakness.

Once found acceptable in all aspects, on a separate day, 20 patients would be assigned to both the trainees for calculating inter-observer variation.

The cut-off to be used for labelling as glaucoma suspect at the camp site in our study would be based on parameters mentioned in Table 3. Any person screened, with any element of any test crossing the threshold would be labelled as suspect.

**Table 3.** Parameters for diagnostic tests for identifying glaucoma suspects.

Investigation	Record	Suspect
ACD	as VH 1-4	VH grade 2 or less
C/D ratio	as 0.3 to total cupping	Ratio of 0.6 or more or asymmetry of more than 0.2
IOP	as mmHg	Recording of more than 22mmHg
FDT	Printout	• More than one spot in central field • One central spot or more than one peripheral spot in disc suspects

 In FDT perimetry reporting, one abnormal spot in the central field or more than one spot in the peripheral field would be considered below the cut-off for normal in this test. Any patient with normal disc examination but abnormal FDT findings will not be labelled as suspected glaucoma but will be advised to undergo detailed visual field analysis in the hospital.

Records of each person screened would be entered Those requiring further investigations would be counselled about glaucoma and importance of early detection. They would be offered a free drop to the base hospital. Those not agreeing to travel on the same day would be given a contact number, in case they would like to report later. Those not reporting within a week would be given a reminder call.

## Conventional detection ^20^

In the case of India, currently no organized community-screening program specifically for glaucoma detection exists. At present, detection of glaucoma is through ‘opportunistic case finding’ of patients who present themselves to various eye clinics in the country for various ophthalmic complaints.

The opportunistic case finding of patients presenting in SCEH would be the comparator group for our study. The inclusion criteria would be patients ≥40 years of age and belonging to the low-paid category in the hospital to match the socioeconomic status in the intervention arm. People with prior history of glaucoma screening in community or clinic or already diagnosed with glaucoma would be excluded from the study. After presentation to SCEH, patients would undergo glaucoma consultation at the hospital.

For those travelling to the hospital, transport and investigations in the hospitals would be offered free of cost. Overnight stay if required would also be provided free. A record would be made of the tests, number of persons transported and number staying overnight for cost analysis.

In the hospital, a detailed workup would be done for the glaucoma suspects, so as to confirm or rule out the diagnosis of glaucoma with the following tests: refraction, applanation tonometry, gonioscopy, automated visual field analysis and nerve fibre analysis. Patients will be classified as normal or with OH, probable glaucoma, or glaucoma as per the criteria in Table 4. After the investigations, they would be examined by a glaucoma consultant masked to the source of patient sent. Classification will be done using the criteria mentioned in Table 4. We would look at efficiency using PPV for individual tests based on standard cut-offs

During their time at the hospital the patients/care-givers would be administered a questionnaire about glaucoma awareness (Annexure 1, extended data) ^23^. After the investigations, they would be examined by a glaucoma consultant, who would make a diagnosis of glaucoma, glaucoma suspect, angle closure disease or impending angle closure requiring intervention, using the criteria mentioned in Table 4. The patient requiring intervention would be counselled and others would be discharged. Those requiring surgery would be offered surgery at fixed rates meant for camp patients needing non-cataract surgery. The patients staying overnight would be offered a free drop and records would be maintained.

**Table 4.** Diagnostic criteria for different patient category ^2, 23– 25^.

Category	Criteria
Healthy subjects	• IOP <21 mm Hg with no history of elevated IOP • Normal appearing optic disc, intact neuroretinal rim, and RNFL • Minimum of two reliable normal visual fields, defined as pattern standard deviation (PSD) within 95% confidence limits and a glaucoma hemifield test (GHT) result within normal limits • No family history of glaucoma
Glaucoma suspects	• Disc suspects: Those who met disc criteria i.e, (structural and functional evidence) Eyes with CDR > 0.6 or CDR asymmetry > 0.2, or neuroretinal rim width reduced to <0.1 CDR between 11 to 1 o’clock or 5 to 7 o’clock, but were not proved to have definite field defects. • Field suspects: Those with definite field defects, but not meeting above disc criteria. • Those with optic disc margin haemorrhages. • Those with an IOP >97.5th percentile(>21 mm Hg) ^24^ • Primary angle closure suspect: An eye in which appositional contact between the peripheral iris and posterior trabecular meshwork is considered possible defined as an angle in which ≥270° of the posterior trabecular meshwork (the part which is often pigmented) cannot be seen • Primary angle closure(PAC): An eye with an occludable drainage angle and features indicating that trabecular obstruction by the peripheral iris has occurred, such as peripheral anterior synechiae, elevated intraocular pressure, iris whorling (distortion of the radially orientated iris fibres), “glaucomfleken” lens opacities, or excessive pigment deposition on the trabecular surface. The optic disc does not have glaucomatous damage ^25^
**Primary open angle** **glaucoma (POAG)**	• ONH changes characteristic of glaucoma (focal or diffuse neuroretinal rim thinning, localised notching, RNFL loss), disc based on stereoscopic evaluation by volk 90 D and reviewed by experienced grader. • Characterstic glaucomatous field defects (Hodapp Parish Anderson criteria) ^26^ • Open angles • IOP >21 mm Hg at time of diagnosis.
**Primary angle closure** **glaucoma (PACG)**	• ONH changes characteristic of glaucoma (focal or diffuse neuroretinal rim thinning, localised notching or RNFL defects) disc based on stereoscopic evaluation by 90 D and reviewed by experienced grader • Characteristic glaucomatous field defects (Hodapp Parish Anderson criteria) ^26^. • An eye in which appositional contact between the peripheral iris and posterior trabecular meshwork is considered possible defined as an angle in which ≥270° of the posterior trabecular meshwork (the part which is often pigmented) cannot be seen. • IOP>21 mm Hg at the time of diagnosis.
**Normal tension** **glaucoma (NTG)**	• ONH changes characteristic of glaucoma (focal or diffuse neuroretinal rim thinning, localised notching or RNFL defects). Disc based on stereoscopic evaluation by volk 90 D and reviewed by experienced grader • Characterstic glaucomatous field defects (Hodapp Parish Anderson criteria) ^26^. • Open angles • History of untreated peak IOP of 21 mm Hg or less ^4, 24^.
**Criteria for Ocular** **hypertension**	• IOP> 21 mm Hg without treatment • Visual Fields normal • Optic disc and retinal nerve fibre layer normal • Gonioscopy: Open anterior chamber angle (exclude intermittent angle closure) • No history or signs of other eye disease or steroid use • Other risk factors: None

## Aim and objectives

The aim of the study is to measure the cost, cost-effectiveness and detection rate of glaucoma screening in cataract camps in rural India conducted by SCEH. The comparator group would be patients who are undergoing assessment for cataract in other such camps conducted by SCEH and diagnosed with glaucoma on their presentation to the SCEH hospital. The comparator will also include patients reporting directly to SCEH for cataract and/or glaucoma assessment.

The specific objectives of the study are:

a) To measure the detection rate of glaucoma screening intervention in cataract camps in urban India compared with opportunistic and passive screening (usual care);
b) Identify, measure and value the health resource use of the intervention;
c) Estimate cost-effectiveness by evaluating the incremental cost per case detected from a quasi-societal perspective over a year’s time horizon.

### Study design

A prospective study of glaucoma detection and cost assessment of the glaucoma screening conducted in the community. The total and incremental costs of the intervention prospectively will be captured from a quasi-societal perspective, measuring programme, provider and household costs.

### Study geography

**Figure 1.**
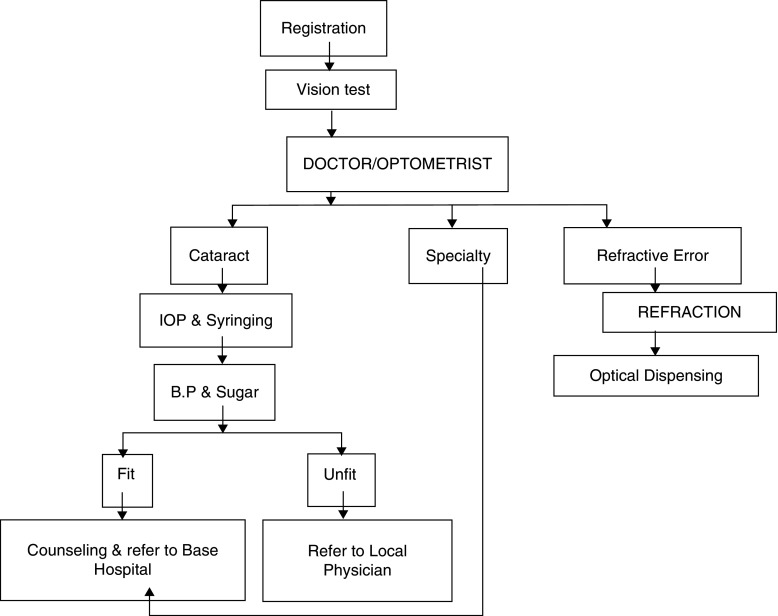
Flow of patients in a routine cataract camp.

**Figure 2.**
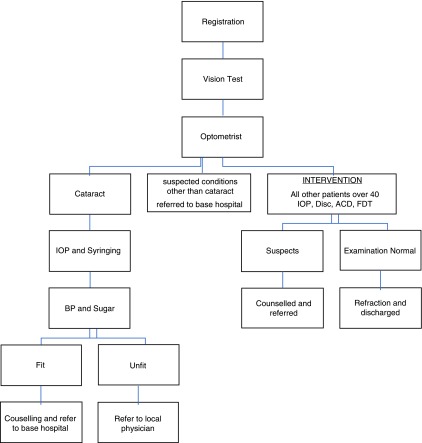
Flow of patients in interventional arm. IOP, intraocular pressure; ACD, anterior chamber depth; FDT, frequency doubling technology.

The camps will be held in urban slums in and around the national capital region of Delhi in North India. Figure 1 and Figure 2 depict the flow of patients, that will be maintained in the routine camps and intervention camps, respectively.

### Study participants

Figure 1 shows the patient flow in a routine cataract camp, whereas, in Figure 2, the patient flow in camps which constituted interventional arm is depicted.

- **A. Intervention arm: From intervention cataract camps**
- All people examined by ophthalmologist with torch and ophthalmoscope.
- Patients aged ≥40 years not having cataract and diagnosed as normal at this step would be included in this arm.

Included patients would undergo ophthalmoscopy, AC depth examination, FDT and IOP measurement with rebound tonometry.

- If identified as neither glaucoma or suspected glaucoma (criteria as per Table 3), they would be discharged from the camp after being labelled as normal.
- If labelled as suspected cases (criteria as per Table 3), referred to hospital.

In hospital, these referred cases will undergo the following examinations:

Undergo complete clinical examination with gonioscopy and 90D examination for disc by a glaucoma specialist.
Detailed visual field examination.
If normal, discharged.
If diagnosed with glaucoma (criteria as per Table 4), advised appropriate treatment (YAG PI, surgery offered free or prescribed medication).
**B. Non-intervention arm: Routine cataract camps**

Those aged ≥40 year old identified from routine cataract camps without any extra equipment or trained optometrist for glaucoma diagnosis:

- People attending camps undergo routine tests by ophthalmologist with torch and ophthalmoscope.
- If diagnosed as having cataract by the ophthalmologist, the subject will undergo IOP with Schiötz tonometry.

Glaucoma suspects ( Table 4) in this arm would come from routine cataract camps if

- detected by ophthalmologist in the camp as suspected glaucoma
- if picked up as cataract from camps, brought for cataract surgery but diagnosed to be having glaucoma after examination in the hospital

**Figure 3.**
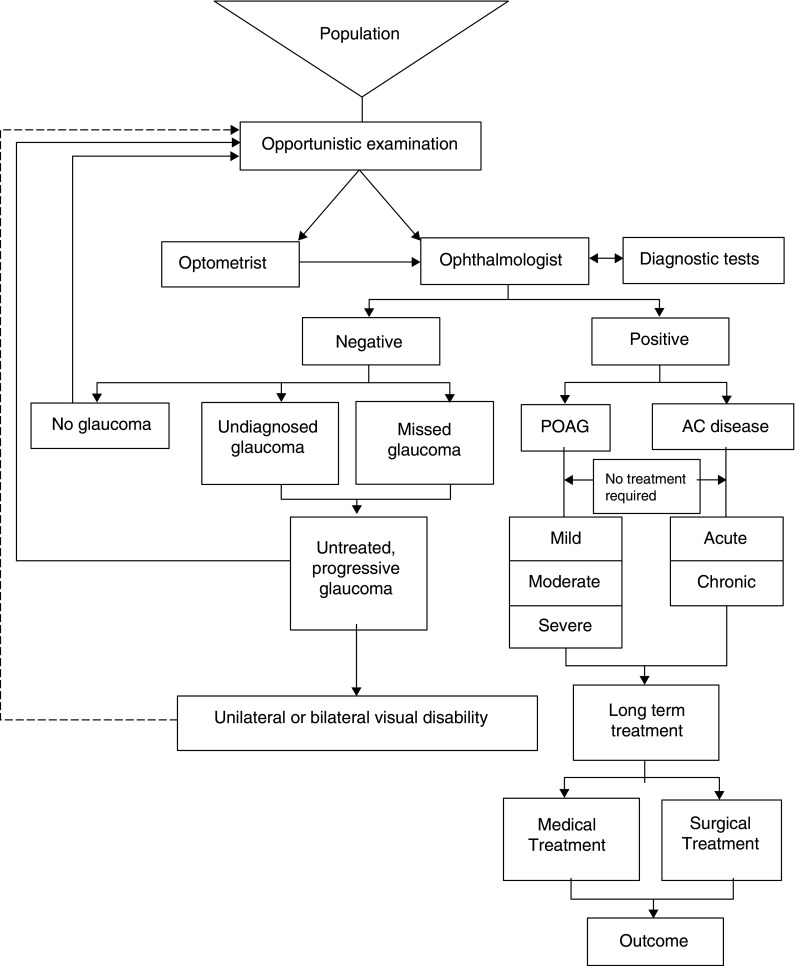
Current diagnostic/treatment approach in India. This figure has been reproduced with permission of John (2011) ^27^.

Undergo tests similar as intervention group in hospital:

- Undergo complete clinical examination with gonioscopy and 90D examination for disc by a glaucoma specialist
- Detailed visual field examination
- If normal, the subject is discharged
- If diagnosed glaucoma--- advised appropriate treatment
- **C. Hospital arm: patients presenting to comprehensive semi-private OPD (with similar socio-economic status to camp patients) ( Figure 3)**

Patients suspected glaucoma for the first time will undergo the following diagnostic tests:

- Examination including gonioscopy with glaucoma specialist
- Visual field test

If diagnosed as glaucoma ( Table 4), appropriate treatment is advised.

### Sample size calculation

The detection rate or yield will be measured by dividing the number of new cases identified during the study by the total number of cases examined. However, various other indicators will also be estimated through the study, which are expected to throw light on the efficacies of the additional setting at the intervention camps in comparison with normal cataract camps and conventional detection setting at the hospital. Therefore, the sample sizes are estimated for different components of the study separately, based on the desired accuracy of the indicators at different level. For an accuracy of ±0.1 with a confidence interval of 95% and assuming a rate of around 50% positive predictive value (PPV) and negative predictive value (NPV), the sample size was estimated to be 97 suspects as diagnosed by the additional setting.

The sample size mentioned above is applicable for both interventional and conventional cataract camps (used in camps set-up A and B) and conventional detection setting (triage) at the hospital set-up C. This sample sizes are estimated using the following formula ^28^:

$$n=\frac{{\text{(}z_{\alpha \text{/2}}\text{)}}^{\text{2}}}{d^{2}}\times population\;variance$$

Where

α is such that (1- α) is the probability of confidence interval

Z _1-α/2_ is defined as probability {|X|< Z _1-α/2_ | X~ N(0,1)} = (1- α)

d = desired accuracy of the estimate. In other words, length of the confidence interval is 2d.

### Null hypothesis

Since the individuals not suspected of having glaucoma in the intervention camps will not be referred to the hospital for confirmatory diagnosis as per the study protocol, the sample for normal population will be captured by the study FDT setting at the hospital set-up B. We assume that only 75% post-screening follow-up compliance rates, i.e. people suspected of having glaucoma, referred from the intervention camps turning up for confirmatory diagnosis. However, the confirmatory diagnosis test can be conducted on all those diagnosed as suspected of having glaucoma in the hospital arm as they will be present in the hospital itself. It has been assumed, for the purpose of estimation, that the rate of detection of people suspected of having glaucoma would not exceed 12% in the community. We further assume that it can be, in reality, as low as 10%. The number of screenings in the intervention, non-intervention and hospital arm should be adjusted accordingly in order to capture the required minimum sample sizes for people suspected of having glaucoma (required for estimating PPV) and normal (required for estimating NPV) populations. Although the setting in the non-intervention arm will also capture a few suspected glaucoma cases, its primary objective is to identify normal individuals to be referred for confirmatory diagnosis, the target for capturing suspects at the intervention camps is reduced. Conventional detection setting in the hospital arm will identify both suspects and normal for confirmatory diagnosis.

Given the above assumptions on (i) post-screening follow-up compliance rates, (ii) prevalent suspect rates for interventional setting and conventional detection setting and (iii) required minimum sample sizes for estimating PPV and NPV, the number of screenings required for different components are estimated as per Table 5. The recruitment of patients across the 3-arms will be conducted until the last patient is recruited as per number of screening required in Table 5.

**Table 5.** Number of screenings required for different components.

Detection method	Assumptions		Number of screening required	Expected outcomes of screening		Lost Follow Up		Turn-ups for confirmatory diagnosis	
	Suspect Rate	Follow-up Compliance Rate		Suspects	Normal	Suspects	Normal	Suspects	Normal
**Studyb** **settings**									
Intervention Camp	10%	75%	**1,150**	115	1,035	29	NA	86	NA
at Hospital	10%	100%	**108**	11	97	0	0	11	97
**Total**			**1,257**	126	1,132	29		97	97
Conventional Detection at hospital	10%	100%	**1,015**	102	914	0	0	97 *	97 *

*Conventional detection setting at hospital would identify many more suspects and normal than what is required to estimate its PPV and NPV. But we need only 97 from each category (suspect and normal). NA, not applicable.

### Study population

All people of age ≥40 years, not detected as having cataract who would consent for screening for glaucoma would be included. A trained optometrist and vision technician would conduct various screening tests, such as disc examination, ACD depth, IOP reading, and FDT. Any result beyond cut-off points as mentioned in Table 3 would be flagged as suspected glaucoma cases and would be received by counselor. For those travelling to the hospital, transport and investigations in the hospitals would be offered free of cost. Overnight stay if required would also be provided free.

In the hospital, a detailed workup would be done for the glaucoma suspects. For glaucoma, they would undergo applanation tonometry, gonioscopy, automated visual field analysis and nerve fibre analysis. After the investigations, they would be examined by a glaucoma consultant, who would be masked to the study arm to which patient belongs. A diagnosis of glaucoma, suspected glaucoma, angle closure disease or impending angle closure requiring intervention will be made. The patient requiring intervention would be counselled and others would be discharged. Those requiring surgery would be offered surgery at fixed rates meant for camp patients needing non-cataract surgery. The patients staying overnight would be offered free transport both to the hospital from the camp and back to the camp site after discharge. Records would be maintained of all patients offered free transport.

### Study design

A prospective observational study of 1258 adults ≥40 years age attending for glaucoma screening in cataract screening camps will be conducted. These adults would be recruited over an 18-month period across camps being conducted in villages near Delhi. The study is ongoing and recruitment is expected to be completed by December 2019. The recruitment would continue till the 97 ^th^ suspect is identified.

The comparator group would be an equal number of adults of similar age who are screened in cataract camps in other nearby similar areas by SCEH and are identified as glaucoma suspects and as glaucoma-positive during their visit to the hospital. Additional comparator would also be patients ≥40 years age of similar socio-economic backgrounds who visit the hospital for the first time as routine or referral patients for eye check-up and are identified as glaucoma suspects and glaucoma positive. 

### Measurement of health outcomes/effectiveness

The primary outcome of the screening algorithm would be the detection rate of the screening tests. The detection rate will be calculated by dividing the number of new cases identified during the study by the total number of cases examined ^27^.

### Cost assessment

We will use a step-down costing methodology for capturing costs of screening camps, whereby program costs will be entered into a customized tool created in MS Excel (Annexure 4, extended data) ^23^. Cost data will be regularly entered into the tool for a period of 3 months to reflect any changes in the cost structure of the screening program. Using a step-down method, the main worksheets for entering data allocate costs to one of the following categories: training, pre-screening and screening.

The costs will be calculated by adding the unit costs of all resources used in the different activities occurring the screening process. This process includes all activities and procedures performed to detect cases with glaucoma from the invitation to participate in the screening campaign to the moment when the ophthalmologist establishes the diagnosis in the hospital. We will use the concept of activity-based costing for the screening program, but for the hospital we will use a standardized cost-accounting system. For the activity-based costing, the costs inputs will include screening invitation, screening costs (health professionals, devices, screening venue etc.). Personnel costs will be calculated from the total cost of each professional participating in a certain activity according to the time specifically dedicated to that particular activity. For example, optometrists dedicated 5–6 hours of their daily work time to the screening and only that part of their full salary at the time of screening will be considered as a cost. For optometrists and ophthalmologists working in the hospital conducting different tasks (training, examination or test interpretation), only the fraction of their work time dedicated to the specific task involved in the glaucoma diagnostic process will be added.

All diagnostic equipment (e.g. Tono-pen, Ophthalmoscope, FDT Perimeter) with a usage life of more than a year will be considered as capital equipment. The life cycle of such equipment will be considered as 2.5 years, and other capital equipments such as slit lamp, trial sets, etc. will be considered for life-cycle of 3 years ^21, 22^. Annual maintenance of all diagnostic equipment will be considered at 10% of annual rental value. Other equipments such as E type Snellen charts, Tumbling E charts will be considered for 1 year life -cycle. Rental value of screening clinics will be captured based on present value based on local information. The overhead costs towards screening (e.g. administrative and management) will be considered at 20% of total running cost ^29^. Any repairs and maintenance of equipment The annual and monthly rental value of the capital equipment, and screening clinic costs per year/month will be calculated as per methods for assessing costs of glaucoma screening in India in other studies ^20, 21^.

Key information interviews with Program Manager (Outreach) will be conducted in identifying any donated goods requiring re-evaluation and in allocating joint costs between program components. The allocation of joint costs to program components and activities, will also be informed by monthly sheets of screening camps.

At the hospital, the costing will focus on estimating the examination cost for glaucoma detection at SCEH. Examination cost will include both direct and overhead costs. Direct costs will include labour, capital and material costs. Labour costs will comprise the salaries and fringe benefits of all staff involved in the examination room, both contractual and regular. The detailed information about the ophthalmologists, optometrists, and support staff involved in each examination room will be taken from the hospital payroll and through confirmation of hospital administrator. For examination room staff who work in other areas, such as operation theatre, wards, and research/teaching (if any), the labour cost will be apportioned based on the working time in each area as reported by the hospital administrator ^30^ Capital costs of the examination room will include; annualised discounted depreciation cost of the building (examination room area), furniture, equipment and instruments used in the examination room and the opportunity cost of the land. Recent government contracts for purchasing equipment, instruments and furniture will be used to capture the price information of capital items. The useful life of building and structures will be considered as 10 years; useful life of furniture and fittings assumed to be 10 years, and that of machinery and plant as 7 years, as per Government of India income tax depreciation rule. However, medical equipment such as FDT, Tonometry etc, the useful life will be considered as 2.5 years. 3% discount rate will be considered to calculate cost of depreciable assets, and interest rate of 1-year government bank fixed deposit rate will be used to calculate the opportunity cost of land ^31, 32^.

The material costs will include drugs, medical supplies, office supplies and utilities (water, telephone, electricity, and internet charges), and will include the actual usage of materials in the examination rooms during the study period. The utility cost will be distributed as per allocation criteria, for example, the electricity cost of the examination room will be calculated on the floor area of the examination room, and water cost will be distributed based on the number of personnel in the examination room. The assignment of other overhead costs, such as administration, nursing administration, laundry, kitchen, maintenance, transport, and store, suitable allocation criteria will be devised based on discussion with the hospital administrator. The simultaneous equation method will be used for overhead cost distribution ^33^. In this method, full adjustment for the interaction of overhead departments and solving a set of simultaneous linear equations will be conducted for allocations to the examination room.

To determine the examination cost, we will first list the equipment needed for each examination and then note the time of usage of each piece. Staff time of ophthalmologist and optometrist for sample of 20 patient examined across the study arms would be captured from interviews with relevant staff. For both staff time and equipment usage, average time will be considered. Each drug and material used for the examination will also be identified as per actual usage for a particular patient. The material cost will be captured from hospital list and material cost for each examination will be conducted.

Costs to the community would be captured using a structured questionnaire and will include direct medical (consultation, medicines etc.), direct non-medical (travel), and indirect costs (wage loss of patient) (See Annexure 2, extended data) ^23^.

### Cost-effectiveness analysis

For the cost-effectiveness analysis, the cost per case detected would be the primary effectiveness outcome measure, in comparison with conventional detection, i.e. opportunistic case finding in hospitals.

### Sensitivity analysis

The costs for the screening program and examination at hospital will be based on the study sample and may vary significantly depending on the size of the target population and other factors. Similarly, detection rate, post-test probability of screening tests, and follow-up screening compliance for further examination at hospital, and costs identified from this study might be different from other studies in similar settings ^20, 21^. A one-way sensitivity analysis will be performed to test the changes to the cost effectiveness results using these parameters which are either a key cost driver, or subject to a high degree of uncertainty.

The costs will also be recalculated for the community of 1 million subjects in order to generalize the findings to India as a whole. Costs will be presented in 2019 prices in Indian rupees and United States dollars (USD). Since the study period is less than 12 months, costs will not be adjusted for inflation, nor will discounting be considered for costs and outcomes.

### Long-term benefits of screening

The long-term benefits and cost-effectiveness of early detection of glaucoma through screening will be analysed using a Markov model, by quantifying the contribution of screening and prevention in reducing the disease related morbidity, blindness and premature mortality in India using data from the current study, relevant published studies, and using expert opinion and assumptions (if any).

### Data entry and storage

Data entry will be conducted by a single data entry operator at the research unit of SCEH. One of the co-authors (AM) will be reviewing the data entry to check for any discrepancies including any data entry errors from the data entry form. The data will be stored in a desktop computer with access to the data entry operator, and co-author (AM). Once the data entry is completed, and cleaned, the data sheet will be transferred to laptops of co-author (AM & DJ) for further analysis. After the analysis these data sheets will be destroyed in these laptops and the data sheet would be available only with the desktop present at research unit of SCEH.

### Ethics approval

The patients being screened would be undergoing standard investigative procedures and no experimental investigation or procedure would be carried out. Informed consent would be taken before administering the questionnaire regarding cost incurred by patient/relative for screening and hospital examination (Extended data, Annexure 3) ^23^. The identity of the person would not be disclosed, and no identifiable data would be shared or published. The identifiable data stored in database would be password protected.

The study received ethical approval from Dr Shroff’s Charity Eye Hospital-Ethics Committee (study reference number SCEHEC/2018/02) on 13.02.18.

### Distribution of study results

The study results will be submitted to a suitable peer-review publication within 6 months of study period (i.e. patients entering camps and then arriving at hospital) with an acceptable sample size as described in relevant section above. Additionally, the results will also be presented in suitable national/international conferences based on resources available for participation.

## Conclusion

This paper constitutes the first published protocol for the cost and cost effectiveness of a glaucoma screening program in a low-middle-income country. The protocol, which will adhere to internationally recognized guidelines for conducting and reporting economic evaluation studies, serves to heighten the transparency of the economic evaluation and planned analyses. The findings from this study will inform funding organizations, eye care institutions and policymakers about the relative value of glaucoma screening in the community. The evidence will contribute significantly to the scarce evidence regarding the cost-effectiveness of community screening for glaucoma in reducing blindness in LMICs.

## Data availability

Extended data for this study, described below, are available on figshare. DOI: https://doi.org/10.6084/m9.figshare.7503887.v4 ^23^.

Annexure 1. Questionnaire concerning glaucoma awareness. English and Hindi versions are given.

Annexure 2. Patient expenses data form.

Annexure 3. Patient informed consent form.

Annexure 4. Customised Excel tool template.

Figures

Data entry template

Data are available under the terms of the Creative Commons Attribution 4.0 International license (CC-BY 4.0).

## Glossary of terms

**IOP**- Intra Ocular Pressure

**ACG** angle closure glaucoma

OAG open angle glaucoma

**C/D-** Cup to Disc ratio, used as one of the signs of glaucomatous damage

**ACD-** Anterior Chamber depth

**FDT**- Frequency Doubling Technology (a type of perimetry used for screening for visual field defect)

**Gonioscopy**- Technique used for visualizing the angle structure, used as gold standard for detecting angle closure

**90D** examination- A diagnostic lens of 90 diopters, used to visualize the optic nerve
