# Supplementary material for: Cost-effectiveness of glaucoma screening in cataract camps versus opportunistic and passive screening in urban India: A study protocol
Source: F1000Res. 2019 Apr 29;8:53. Originally published 2019 Jan 14. [Version 3] doi: 10.12688/f1000research.17582.3 (PMC6518442; doi:10.12688/f1000research.17582.3)
Supplement: Peer review history

# Peer review: Reviewer response for version 3

**DOI:** 10.5256/f1000research.20747.r47800

**Published:** 2019-05-10

**Authors:** Subhash Pokhrel

**Affiliations:** 1 Health Economics Theme (HERG), Institute of Environment, Health and Societies, Brunel University London, London, UK

This refers to version 3 which is based on my earlier comments. The authors have considered my suggestions and implemented most of those in this version.

I have read this submission. I believe that I have an appropriate level of expertise to confirm that it is of an acceptable scientific standard.

# Peer review: Reviewer response for version 2

**DOI:** 10.5256/f1000research.19994.r44888

**Published:** 2019-03-29

**Authors:** Subhash Pokhrel

**Affiliations:** 1 Health Economics Theme (HERG), Institute of Environment, Health and Societies, Brunel University London, London, UK

This article describes a protocol of a study that aims to establish the cost-effectiveness of a glaucoma screening intervention compared with opportunistic screening (in cataract surgery camps) as well as with passive detection at secondary care settings in urban India from a quasi-societal perspective. The study design seems appropriate given the context and the protocol is written up well. However, the following weaknesses in this version of the paper need to be addressed.  

- Simplify the title by saying, “Cost-effectiveness of glaucoma screening in cataract surgery camps vs. opportunistic and passive screening in urban India: a study protocol”
- Abstract: Reorganise as a structured abstract following CHEERS guideline or use standard structure, e.g. Objective, design, setting, participants, intervention, comparator, outcomes, methods (including perspective and time horizon), ethics, etc.
- Introduction and methods texts: clarify the decision rule for suspect detection. Is it one element of any test crossing the threshold or all elements of any test, or all elements of all tests? This is not clear. Also, state in the analysis whether you would look at efficiency in relation to what element of what test has the most predictive power and hence is likely to be cost-effective.  
- Rationale: state clearly that it is helpful for policymakers to understand the rate at which the intervention becomes cost-effective; currently no such data is available. Include this ‘threshold analysis’ in your methods section too.  
- Aim of the study: simplify this by changing the order, e.g. The aims of this study are to: (a) measure the detection rate of a glaucoma screening intervention in cataract camps in urban India compared with opportunistic and passive screening (usual care); (b) identify, measure and value the health resource use of the intervention; and (c) estimate cost-effectiveness by evaluating the incremental cost per case detected from a quasi-societal perspective over a year’s time horizon.
- Study design – this does not appear to be strictly a ‘cohort’ study as you are not following up those who are negative on screening and it is less clear whether you would follow up those ‘suspects’ who are negative on hospital examination (some of who may be false negative and false positives at both stages- less clear whether you are capturing those opportunity costs here). Clarify this in the texts. You may want to refer this design just as a ‘prospective study’.
- Sample design – inconsistent across the texts. 97 is the number of suspects and 1000 is expected numbers to be screened (Table 5 provides other numbers). Rewrite the texts to add clarity.
- Recruitment – what is the stopping rule? Would you stop as soon as you have 1000 ^th^ patient or you would continue until you get the 97 ^th^ suspect? Not clear.
- Costing methods: Please provide a statement confirming your costing method is consistent with ‘opportunity cost’ concepts. State explicitly that, for instance, optometrists who dedicate 5-6 hours in the intervention would do other work outside this time (hence your current assumption about including that part of their salary).
- Costing assumptions – rationalise these either by explaining or referring to earlier work/current practice, e.g. why 2.5 years and 3 years of life cycle; 29% overhead?
- Sensitivity analysis – please list all key assumptions here that would be subject to sensitivity analyses and justify.
- Long term costs and benefit – this was picked up by reviewer 1 and I have seen the author’s response to this. Modelling long term cost savings – I guess - would be a marginal effort with much higher anticipated return in terms of the value of this information to policymakers. This would be a missed opportunity – suggest you reconsider this if you can.
- Annexes – the costing template looks to be a good start but would benefit from adding specific details around types of health resource. Consider expanding this in the next version. Likewise, patient expenditure questionnaire would benefit from having close questions rather than open ended question on resource use.
- Perspectives – perhaps say quasi-societal as you are not capturing inter-sectoral costs and benefits?  

I have read this submission. I believe that I have an appropriate level of expertise to confirm that it is of an acceptable scientific standard, however I have significant reservations, as outlined above.

# Peer review: Reviewer response for version 2

**DOI:** 10.5256/f1000research.19994.r44864

**Published:** 2019-02-26

**Authors:** Anil Gumber

**Affiliations:** 1 Faculty of Health and Wellbeing, Sheffield Hallam University, Sheffield, UK

All my comments have been adequately addressed, so I approve the revised version.

I have read this submission. I believe that I have an appropriate level of expertise to confirm that it is of an acceptable scientific standard.

# Peer review: Reviewer response for version 1

**DOI:** 10.5256/f1000research.19226.r43006

**Published:** 2019-01-30

**Authors:** Anil Gumber

**Affiliations:** 1 Faculty of Health and Wellbeing, Sheffield Hallam University, Sheffield, UK

I have made editing comments on the PDF file.

 The protocol is well written.

 Some editing comments e.g. for a couple of tables the title is misleading. Also at some places the population considered for screening mentioned as over 40 years whereas in most places including in tables/figures >= 40. This needs to be corrected.

 At several places abbreviations are used, so it would be useful to provide a glossary at the end of the paper. Also, the term "Icare" is used without defining it properly.

 In the introduction paragraph, the authors have not provided difference in two types of glaucoma and why one's share is greater then other in India and whether their share is consistent with other LMICs.

 In the methods section there are problems in calculating various costs at the organisation level as well as at the patient level. For patient level, the out-of-pocket expenditures on medical equipment/aids has not been considered due to glaucoma care.

 The training cost to field staff has been considered but their validation of enhanced skills for correct detection of glaucoma screening has not been elaborated.

 At organisation level, there is not enough clarity about calculating the unit cost and portioning of staff time used for screening. Multiple activities of staff and multi-usages of fixed cost items such as equipment, furniture and buildings as well as variable cost items (e.g. drugs and consumables) has not clearly been spelt out. Salary cost does not include the employer contribution to pension, earned/casual leave etc. and thus staff cost to the organisation turned out to be 40-60% higher than salary alone. The overhead cost (e.g. admin and management - usually 29% of medical personnel cost) has not been fully explained. Repairs and maintenance of equipment and depreciation for equipment/furniture/vehicle are not explained. Most importantly, the capital cost in terms of building and land (rental value) has not been considered at all.

 The opportunity cost of buildings and equipment used in the production of services, we need to have an estimate of the costs involved and to make assumptions about both the length of time that the ‘investment’ will be tied up in the service, and the rate of return on that investment. In public health services ‘long-run marginal opportunity cost’ is focused, which is the cost of supporting one extra patient while recognising the financial implications of necessary expansion to the service. In the UK, a buildings’ useful life is considered 60 years thus the annuitizing rate is 2.5% (i.e. converting a capital investment into the annual equivalent cost for the period over which the investment is expected to last).

 In terms of cost and cost-effective analysis, the paper has not provided enough details including methods of smoothing the costs data. The long-term benefits of early detection of glaucoma through screening has not been elaborated and modelled. It is important to bring into cost-effectiveness framework by quantifying the contribution of screening and prevention in reducing the disease related morbidity, blindness and premature mortality.

 The inclusion of above comments will definitely improve the quality of the protocol.

I have read this submission. I believe that I have an appropriate level of expertise to confirm that it is of an acceptable scientific standard, however I have significant reservations, as outlined above.

# Response

**Published:** 2019-02-12

**Authors:** shalinder sabherwal

**Affiliations:** shalinder.sabherwal@sceh.net, India

Response to Reviewer 1

**Comment**: The protocol is well written.

**Response**: Much thanks for this comment

**Comment**: Some editing comments e.g. for a couple of tables the title is misleading. Also at some places the population considered for screening mentioned as over 40 years whereas in most places including in tables/figures >= 40. This needs to be corrected.

**Response**: We have corrected the tables. We have also used > 40 years uniformly across all sections of the manuscript.

**Comment**: At several places abbreviations are used, so it would be useful to provide a glossary at the end of the paper. Also, the term "Icare" is used without defining it properly.

**Response**: We have added a section on Glossary of terms at the end of the paper. Details of Icare is now inserted as foot note.

**Comment**: In the introduction paragraph, the authors have not provided difference in two types of glaucoma and why one's share is greater then other in India and whether their share is consistent with other LMICs.

**Response**: We have now inserted these sections in the introductory paragraphs.

**Comment**: In the methods section there are problems in calculating various costs at the organisation level as well as at the patient level. For patient level, the out-of-pocket expenditures on medical equipment/aids has not been considered due to glaucoma care.

**Response**: We have detailed section of organisational costs. Patient costs including out-of-pocket expenditure including drugs have been inserted in Annexure 2. 

**Comment**: The training cost to field staff has been considered but their validation of enhanced skills for correct detection of glaucoma screening has not been elaborated.

**Response**: Validation of skills is mentioned in the training section of the manuscript.

**Comment**: At organisation level, there is not enough clarity about calculating the unit cost and portioning of staff time used for screening. Multiple activities of staff and multi-usages of fixed cost items such as equipment, furniture and buildings as well as variable cost items (e.g. drugs and consumables) has not clearly been spelt out. Salary cost does not include the employer contribution to pension, earned/casual leave etc. and thus staff cost to the organisation turned out to be 40-60% higher than salary alone. The overhead cost (e.g. admin and management - usually 29% of medical personnel cost) has not been fully explained. Repairs and maintenance of equipment and depreciation for equipment/furniture/vehicle are not explained. Most importantly, the capital cost in terms of building and land (rental value) has not been considered at all.

**Response**: We have mentioned screening time per patient section which details the time of optometrist and receptionist time per patient for screening is now inserted. We have also added sections to capture salary, fringe benefit of optometrist, receptionist and helper. Overhead cost of 29% has been inserted. Annual maintenance costs of diagnostic equipment at 10% of annual rental value has been considered. Rental value of screening clinic as per local information has been inserted.

**Comment**: The opportunity cost of buildings and equipment used in the production of services, we need to have an estimate of the costs involved and to make assumptions about both the length of time that the ‘investment’ will be tied up in the service, and the rate of return on that investment. In public health services ‘long-run marginal opportunity cost’ is focused, which is the cost of supporting one extra patient while recognising the financial implications of necessary expansion to the service. In the UK, a buildings’ useful life is considered 60 years thus the annuitizing rate is 2.5% (i.e. converting a capital investment into the annual equivalent cost for the period over which the investment is expected to last).

**Response**: We have used opportunity cost of buildings and equipment as per standards in Indian settings, including life-cycle and discounting considerations.

**Comment**: In terms of cost and cost-effective analysis, the paper has not provided enough details including methods of smoothing the costs data.

**Response**: Smoothing of costs data has been considered using annualised costs, including simultaneous equation method for overhead cost distribution.

**Comment**: The long-term benefits of early detection of glaucoma through screening has not been elaborated and modelled. It is important to bring into cost-effectiveness framework by quantifying the contribution of screening and prevention in reducing the disease related morbidity, blindness and premature mortality.

**Response**: Our objective of the study is to capture the cost per detection rate across the intervention and comparator arms. We have not considered the long-term benefits of early detection of glaucoma and modeling as 2 studies in urban and rural India using decision analytical model have been considered by John & Parikh (2017), and John & Parikh (2018), and mentioned in references. However, as a follow-up we might consider building a Markov model based on study results to estimate the long-term benefits of glaucoma screening in India.

The inclusion of above comments will definitely improve the quality of the protocol.
